# Fairness and legitimacy of decisions during delivery of malaria services and ITN interventions in zambia

**DOI:** 10.1186/1475-2875-9-309

**Published:** 2010-11-01

**Authors:** Mary Tuba, Ingvild F Sandoy, Paul Bloch, Jens Byskov

**Affiliations:** 1 Center for International Health, University of Bergen, PO Box 7804, N-5020 Bergen, Norway; 2 DBL - Centre for Health Research and Development, Faculty of Life Sciences, University of Copenhagen, Thorvaldsensvej 57, DK 1871 Frederiksberg, Denmark; 3 Steno Health Promotion Center, Steno Diabetes Center, Niels Steensens Vej 8, DK-2820 Gentofte, Denmark

## Abstract

**Background:**

Malaria is the leading cause of morbidity and the second leading cause of mortality in Zambia. Perceptions of fairness and legitimacy of decisions relating to treatment of malaria cases within public health facilities and distribution of ITNs were assessed in a district in Zambia. The study was conducted within the framework of REsponse to ACcountable priority setting for Trust in health systems (REACT), a north-south collaborative action research study, which evaluates the Accountability for Reasonableness (AFR) approach to priority setting in Zambia, Tanzania and Kenya.

**Methods:**

This paper is based on baseline in-depth interviews (IDIs) conducted with 38 decision-makers, who were involved in prioritization of malaria services and ITN distribution at district, facility and community levels in Zambia, one Focus Group Discussion (FGD) with District Health Management Team managers and eight FGDs with outpatients' attendees. Perceptions and attitudes of providers and users and practices of providers were systematized according to the four AFR conditions relevance, publicity, appeals and leadership.

**Results:**

Conflicting criteria for judging fairness were used by decision-makers and patients. Decision-makers argued that there was fairness in delivery of malaria treatment and distribution of ITNs based on alleged excessive supply of free malaria medicines, subsidized ITNs, and presence of a qualified health-provider in every facility. Patients argued that there was unfairness due to differences in waiting time, distances to health facilities, erratic supply of ITNs, no responsive appeal mechanisms, inadequate access to malaria medicines, ITNs and health providers, and uncaring providers. Decision-makers only perceived government bodies and donors/NGOs to be legitimate stakeholders to involve during delivery. Patients found government bodies, patients, indigenous healers, chiefs and politicians to be legitimate stakeholders during both planning and delivery.

**Conclusion:**

Poor status of the AFR conditions of relevance, publicity, appeals and leadership corresponds well to the differing perceptions of fairness and unfairness among outpatient attendees and decision-makers. This may have been re-enforced by existing disagreements between the two groups regarding who the legitimate stakeholders to involve during service delivery were. Conflicts identified in this study could be resolved by promoting application of approaches such as AFR during priority setting in the district.

## Background

Malaria is a threat to more than 40% of the world's population [1]. Approximately 50 million pregnant women are exposed to malaria each year [2,3]. Of all the annual malaria cases in the world, 90% occur in sub-Sahara Africa, and the majority (75%) of these cases are in children <5 years of age. Moreover, an estimated 74% of the populations in sub-Saharan Africa live in areas that are endemic to malaria [3]. Furthermore, 80% of malaria in the region is managed at home [4].

Malaria is the leading cause of morbidity and the second leading cause of mortality in Zambia. Malaria transmission occurs in almost all parts of the country and accounts for up to 40% of the overall infant mortality rates and up to 20% of the overall maternal mortality rate [5]. Transmission takes place from November to May. A high proportion (around 45%) of hospitalizations and outpatient department visits are attributable to malaria. Malaria incidence and death rates have tripled over the past three decades, but in the period 2003 to 2007, most provinces had a downward trend in malaria incidence, and nationally the incidence dropped from 425 per 1,000 in 2006 to 358 per 1,000 in 2007 [5]. This may have been a result of the impact of aggressive efforts towards implementation of the nationally prioritized malaria control strategies, i.e insecticide-treated nets (ITNs), case management, intermittent presumptive treatment (IPT) and vector control, specifically in-door residual spraying (IRS). This paper focuses on malaria treatment within health facilities (case management) and ITN distribution as the top two of the four prioritized malaria control strategies.

Malaria Control priority setting processes typically have a top-down approach, starting at the World Health Organization (WHO) Global Malaria Control Programme (GMCP). Global strategies are being brought to continental attention through partnerships and declarations such as the Multi-lateral Initiative on Malaria (MIM) and the Abuja declaration of 2000, respectively. Priorities are then adopted for implementation by member countries of WHO regions. The Roll Back Malaria (RBM) initiative, which is a WHO strategy, aims to reduce the global malaria burden on one hand, and strengthen health systems on the other by improving efficiency and effectiveness as well as strengthening implementation of prioritized malaria control activities [6].

In Zambia, prioritization of healthcare services, particularly malaria services and ITN interventions, are done through partnerships constituted of diverse stakeholders, including Ministry of Health (MoH) heads of departments and units, other governmental institutions and line ministries, statutory bodies, academic and training institutions affiliated to MoH, Civil Society Organizations (CSOs), Non-governmental Organizations (NGOs) and other private health institutions, multilateral and bilateral cooperating partners [7]. The top-down prioritization processes continue down to the district level, where each district contextualizes the priorities and should revert to a bottom-up approach [8]. In line with partnerships building recommended by the Roll Back Malaria initiative and the national guidelines for priority setting, each district should involve those stakeholders recognized and accepted by formal decision-makers and informal decision-makers. These latter stakeholders should include informal decision-makers such as Community Health Workers (CHWs), Neighbourhood Health Committees (NHCs), Health Centre Committees (HCCs) including their malaria agents (community volunteers specifically recruited to implement malaria control activities), indigenous healers and chiefs. On the other hand, formal decision-makers involved at district level should include donors, non-governmental organizations (NGOs), heads of departments from other governmental or quasi-governmental institutions, politicians, renowned business houses or individuals supporting health activities in the district etc. However, the final technical decisions are made by the DHMT management.

Health facilities receive feedback on the satisfaction of the population with the services provided through suggestion boxes, where patients and other community members may post their complaints, compliments or appraisals. According to national guidelines these submissions should ideally be read by formal decision-makers, and whenever major complaints are identified, a meeting with the community should be called where possible solutions, including community assistance, may be discussed.

The policy guidelines of the Zambian Government relating to malaria drugs states that malaria drugs should be free of charge, especially in areas classified as rural where user fees have been abolished. In addition, subsidized ITNs should be accessible through the Antenatal Clinics (ANC) and under-five-children clinics since children <5 years and pregnant women are prioritized higher risk groups. Distribution of subsidized ITNs at community level is done through community-based partnership arms such as malaria agents, Neighbourhood Health Committees (NHCs) and Community Health Workers (CHWs).

The REsponse to ACcountable priority setting for Trust in health system (REACT), is a North-South collaborative multi-centre action research study, which aims to strengthen the legitimacy and fairness of priority setting at district level in Zambia, Tanzania and Kenya [9]. Delivery of malaria services and distribution of ITNs was one of the areas addressed, to obtain in-depth insight into priority setting within programmatic services and interventions.

### Origin and rationale of AFR

The nature, purpose and function of the AFR framework are to support or guide fair and legitimate decision-making (process) and decision-taking (carrying it out) during priority setting and allocation of healthcare resources within institutions. Having developed from research on allocation of healthcare resources, the AFR was initially formulated as a tool for priority setting and rationing. It has been acknowledged that resource allocation decisions in healthcare were rife with moral disagreements and a fair, deliberative process was necessary to establish the legitimacy and fairness of such decisions [10]. This framework argues that in the absence of consensus on principles, a fair process allows us to agree on what is legitimate and fair. Key elements of fair process involve transparency about the grounds for decisions, appeals to rationales that all can accept as relevant to meeting health needs fairly; and procedures for revising decisions in light of challenges to them [11].

This paper looks at, among other things, priority setting and rationing at the individual level during delivery of malaria services. Health systems research always has an institutional-individual link (macro-micro link) in the sense that encounters, experiences, observations, perceptions, knowledge relating to fairness and legitimate decisions, attitudes and practices of providers and users are continuously being shaped by existing social environment and processes upon and within which malaria services in particular occur. Health providers are individuals within a health institution and are, therefore, influenced by the workload, staffing, work culture and other principles, which are upheld as important within the health institution they work. Thus the behaviour of individual health providers may be seen as an indicator of the principles, or the lack of principles, guiding priority setting in an institution. Patients on the other hand are individuals also using healthcare services within an institution. Since the AFR emphasizes "institutional process" [12], the AFR framework with its conditions was appropriate to answer the research questions. The objective of this study was to assess local perceptions of fairness and legitimacy of decision making related to the delivery of malaria services at district level in Zambia. In order to identify how the perceived fairness and legitimacy of priority setting could be improved, calls for improvement were categorized according to the AFR condition relevance, publicity, appeals and leadership.

### Methodology

In this paper, the use of the Accountability For Reasonableness (AFR) ethical framework was explicit when systematically identifying and assessing attitudes, perceptions and practices which, were reflecting the fairness and legitimacy of decision-making related to delivery of malaria services (treatment of malaria cases within public health facilities) in public health institutions and ITN distribution in a rural district in Zambia. Individual health providers, patients and other community informants were interviewed about their perceptions of whether or not malaria services delivered or distribution of ITNs were fair and, whether planning and delivery of malaria services and ITN distribution involved legitimate stakeholders. Perceptions, attitudes and practices of providers and users were systematized according to the four AFR conditions relevance, publicity, appeals and leadership.

This study acknowledges that aspects of the four AFR conditions do not exist independently, but are closely associated with other underlying principles such as those found in equity, quality and trust. Trust in the REACT project is considered a proxy indicator for legitimacy, accountability and responsiveness, and where utilization is associated with trust in health systems [9]. Equity on the other hand, expresses equal opportunities e.g. for receiving services. It has been defined as the absence of systematic disparities in health between groups with different levels of underlying social advantage/disadvantage--that is, wealth, power, or prestige [13]. Quality on the other may include technical and managerial aspects as defined by providers, perceptions of responsiveness, satisfaction, respect and effectiveness from the user perspective. This study therefore, views the 4 AFR conditions as embracing these three when assessing them.

## Methods

Between 2006 and 2008, in-depth interviews (IDIs) and focus group discussions (FGDs) were conducted in Kapiri-Mposhi district to provide baseline data for the REACT project. This was part of the REACT cross-sectional baseline study employing both quantitative and qualitative techniques of data collection. Recruitment and interviewing of informants and participants was limited to and representative of 45 randomly selected Standard Enumeration Areas (SEAs) located within the demographic boundaries known as Census Statistical Areas (CSAs).

### Description of study site

Kapiri-Mposhi is predominantly a rural district of Zambia, which is located in the Central province. It is a gateway to the north of the country, with major roads connecting to Tanzania and the Copperbelt province in the north-west direction. The population of this district stood at approximately 273, 147 in 2006 with the growth rate of 5.8% - *Central Statistics Office census 2002 extrapolated for 2006 *[14]. The major economic activity is subsistence and limited commercial farming.

Kapiri-Mposhi district has 20 public health facilities, five health posts, four private and two mission health facilities spread across the district [15]. A 2^nd ^level referral hospital located in a nearby Kabwe district serves referrals from Kapiri-Mposhi district who needed services resulting from accidents, X-rays and those with maternal health complications. Infant mortality rate in 2006 stood at 106/1000 in comparison to maternal mortality rate which was 625/100, 000. At district level, malaria was the leading cause of morbidity and mortality. According to information from the Health Management Information System (HMIS) data for Kapiri-Mposhi district, malaria incidence rates varied in 2007 from 104.7 per 1,000 in the first quarter to 55.3 per 1, 000 in the forth quarter [16].

During the period of data collection, ITN distribution was carried out by international NGOs, including the Society for Family Health and Care International, and the District Director of Planning (DDP) through the District Water and Sanitation Health (DWASHA) programme. In addition, Medicine Sans Frontiers (MSF) Greece (defined as donor) was involved in both distributing ITNs and the Intermittent Presumptive Treatment (IPT) programme for pregnant women.

### Sampling procedures for recruiting informants and participants

Eligible informants were identified by employing a matrix which specified desired characteristics such as position and level in hierarchy within an institution and at what level (district, NGO/donor, facility or community). They should occupy decision-making positions and be involved in rationing healthcare resources and in priority setting processes relating to healthcare services, including malaria-related services and interventions. Positions were sampled and not individuals. Thirty-eight (38) in-depth interviews (IDIs) were conducted with decision makers. Nine (9) focus group discussions (FGDs) consisting of 6-10 participants were also conducted with one group of DHMT managers, two groups of male outpatients (aged 15-34 years and 35 years and above), two groups of female outpatients (aged 15-34 years and 35 years and above), two groups of antenatal women (aged 15-34 years and 35 years and above), and one group each of male and female adolescent outpatients aged 18-24 years (Table 1). Thus the community participants all had experience with health service delivery at the facilities and could report on users' perspectives of priority setting in the district and at the facility-level. In order to minimize recall and recognition biases in reporting of experiences relating to fairness and legitimacy of decisions, participants for the outpatient FGDs were recruited immediately after utilizing services within public health facilities. Health providers working in various departments and units of facilities assisted in recruiting participants.

**Table 1:** Distribution of informants and participants by level

Level of data collection	Total number of IDIs	Total number of FGDs
District level (DHMT)	15	1
Facility	10	
Community	13	8
**Total**	**38**	**9**

### Content of guides for In-depth Interviews and Focus Group Discussions

Topics in the IDIs and FGDs guide included definitions, views and experiences related to priority setting as well as potential improvements expected from applying concepts such as relevance, publicity, appeals/revisions and leadership/enforcement during priority setting of disease programmes and service provision. In addition, the guides explored concepts, such as fairness, equity, quality and trust relating to malaria services and ITN distribution. Experiential examples were sought throughout interviews and discussions.

1) Were providers transparent enough to patients and other community informants whereby they could provide grounds upon which decisions made at different points of service use? Did arriving at a decision consider encompassing shared and compromising on values, principles and criteria for priority setting as well as the wider involvement of stakeholders?

2) When patients and other community informants' challenged a particular decision and called for its revision during delivery, how did providers respond?

3) Were there mechanisms to challenge decisions made during delivery?

4) What characteristics did leaders (health providers) working within health institutions possess? Where mechanisms available, which leaders could use to enforce shared values and principles during delivery of malaria services and distribution of ITNs?

All interviews and discussions were tape-recorded, transcribed verbatim and typed to allow for either electronic or manual analysis.

### Procedures for analyses

Analyses were manually carried out using the *"code sheet"*, which is an interpretative understanding approach [17]. Interpretative understanding approach refers to drawing interpretation of findings based on responses provided by informants or participants (emic view). The implications are that what is presented represents original and a true reflection of informants' and participants views relating to fairness and legitimacy. A *"code sheet*", which contained all malaria phrases from IDIs and FGDs was created.

Manual analysis was two-staged as follows:

1) All malaria phrases which were describing fairness or legitimate stakeholder involvement during priority setting processes relating to malaria services and ITN distribution were identified.

1a) Standard codes were assigned to all phrases, which were represented by fairness and legitimacy. The frequency and occurrence of common phrases according to the code were noted.

2) All phrases representative of the AFR conditions relevance, publicity, appeals/revisions and leadership/enforcement were assigned appropriate sub-codes (Table 2).

**Table 2:** Accountability for reasonableness analytical framework

AFR condition	Explanation
**Relevance**	Rationales for priority setting decisions must rest on reasons (evidence and principles) that 'fair-minded' people can agree are relevant in the context

**Publicity**	Priority setting decisions and their rationales must be publicly accessible - justice cannot abide secrets where people's well being is concerned

**Revisions/appeals**	There must be a mechanism for challenge, including the opportunity for revising decisions in light of considerations that stakeholders may raise

**Enforcement**	There is either voluntary or public regulation of the process to ensure that the first three conditions are met.

(Source: Daniels and Sabin, 2002)

2a) The sub-coding was done in order to ascertain the contribution of each of the 4 AFR conditions to definitions of fairness and legitimate. In order to ascertain the density of calls requiring priority improvements according to the AFR conditions, the frequencies and occurrences of phrases were noted.

To check for intra and inter-coder reliability of phrases at different stages of analyzes, transcripts were read and re-read. Inter-coder reliability check meant that when coding of one IDI was completed, all other IDIs and FGDs were read in order to place the same code in other transcripts. This meant that once a code was assigned to a phrase and was placed under an appropriate code, the principle author repeatedly read through single IDIs, FGDs in order to note the frequency and occurrence of similar or same phrases, and then cross checked this across all the IDIs and FGDs.

### Ethical considerations

Clearance to carry out the research was firstly obtained from the Centre for Health Research and Development (DBL), in Denmark. In Zambia, further ethical clearance was obtained initially from the ethical committee of the University of Zambia, School of Medicine, in Lusaka. Additional clearance was obtained from Ministry of Health (MoH). Consent, authorization and authentication of the research was also obtained from the provincial and district health offices. Appropriate and recommended consent procedures to carry out interviews and discussions with individuals and groups at the district, facility and community levels were adhered to throughout the study. At community level, this implied approaching indigenous leaders as well as other influential leaders to request permission to carry out interviews with them and their subjects. Finally, permission to use REACT data was obtained from the Scientific and Steering Committees of the REACT project. This entailed writing a letter of request were the applicant submits documents containing information on the period within which the data would be used, potential co-authors and their institutional affiliations, tentative title of a protocol or publication, objectives and proposed analytical framework.

## Results

Twenty-five interviews with decision-makers at district- and facility-level were carried out. Since eight of the nine participants who took part in the district and facility level focus group discussion with decision-makers also were interviewed using the in-depth interview guide, the total individual contributions from formal decision-makers at district and facility level were 26. Representing the community level, 13 informal decision-makers were interviewed and 52 outpatients participated in the focus group discussions, i.e. individual contributions were obtained from 65 community informants and participants.

### Socio-demographic characteristics of informants and participants

The median age for formal decision-makers group was 38 years (age range 24 to 54 years). Median length of stay in the district was 6.5 years (range 1 to 21 years) and median length of service in the district was six years (range 2 to 29 years). There was also a great variation in their qualifications: accountant, procurement officer, clinical officers, nurses, sociologist, laboratory technologists and physiotherapist. The median age of community decision-makers was 43 years (range 29 to 72 years). Median length of stay in the district was 4.5 years (range 4 to 35 years) and median length of service as a community decision-maker was 5 years (4 to 27 years). The median age for patients was 27.5 years (16 to 76 years) and they had stayed in the district for a median length of 10 years (range 6 months to 35 years).

### Fairness during delivery of malaria services

There were disagreements between formal decision-makers, community (patients) and informal decision-makers regarding fairness during delivery of malaria services. Decision-makers argued that there was fairness in the delivery of services and distribution of ITNs based on availability of free malaria medicines and subsidized ITNs, the high number of people using their services, diverse stakeholder support of malaria services and ITN distribution, and presence of at least one qualified health-provider in facilities or hospital wards. Unfairness according to most formal decision-makers was predominantly looked at from a technical point of view whereby a health facility may lack equipments such as X-ray machines and facilities like theatre room.

*"Of course we are looking at delivery of health services. When you compare this hospital with others, you would find that other certain things are not here which are supposed to be here. I think that's not fair in delivery of health services. Kapiri is quite really a very dense populated area; you know we have to depend on other hospitals like Kabwe to refer. I think that's not also fair on the part of those people. So I think fairness is when things are balancing, when things are not balancing then that is taken as not being fair" *(Facility level decision-maker, male informant).

Informal decision-makers at community level emphasized non-technical aspects when defining fairness.

*"In general, it depends on a situation. Maybe it's fairness in terms of the age. How fair are you during provision of service? What criteria do you follow? But as a human being you find that you may not be very fair. What I understand by fair is how accommodative are you during provision. Do you look at their walks of life, or their status in life? Or just generally, do you offer it on first come first serve" *(Community level decision-maker, male informant).

### Health providers' presence, attitudes and practices

When asked to comment on equity, quality and trust related to malaria service delivery and ITN distribution, the community participants stated that there was unfairness. They gave examples of uncaring, harsh and cynical attitudes by health providers, discrimination, unwelcoming reception approaches (such as neglecting the principle of first-come first-serve and emergencies first), differences in waiting time at the facilities, and lack of responsive appeal mechanisms (e.g. response to complaints made by patients), access to and adequacy of malaria medicines, lack of accountability relating to availability of malaria medicines and subsidized ITNs. Patients (49 of 52 participants) also complained about inadequate number of qualified health-providers to carry out quality case diagnosis and said they suspected that health providers were not present at work during their working hours.

*"There are no doctors in most of the rooms, so every time we come here, it's congestion. They do not perform their jobs to satisfaction. Nakumukoshi kulafita (literally translated as "even my throat becomes dark"). No desire (referring to herself) to receive a particular service despite going to the hospital." *(participant, G6 schooling: FGD with outpatient females 35 years and above)

### Differential treatment of patients

Decision-makers argued that it was fair that waiting times differed for different types of people. When asked, *"Are there any people who are complaining that you favour others, like the haves and the have-nots?" *one formal decision-maker answered thus:

*"Well in society, we have different people. Like even politicians can't go in the queue. So that's how you find, when people see that, they will start complaining. But it's because maybe of one's status in society, for example, the xxx (referring to a political position in the district) and other political leaders"*. (FGD with decision-makers district level, male participant).

The justification by formal decision-makers was that stakeholders such as politicians held influential positions (status) in society and therefore, could not be allowed to stand in queues like any other *'ordinary' *person. Politicians' time was regarded as more valuable than other patients. One decision-maker also mentioned that politicians could make life difficult, e.g. by having a health provider transferred to another place, if they were not satisfied with the services provided, so it was better to do whatever needed to make them content:

*"I have talked of various types of leadership. For example, I will give an example of maybe us as a district here. We want to distribute the rural health centre kits and then probably the xyz (politician) phones the head of department and says I want that truck today because I want to get my fertilizer from some where, now you may find that probably because you feel that you will be regarded as disobedient or what, you find that has an impact now, the distribution of kits would be delayed, postponed to another time, meanwhile people want drugs in the rural health centres. Political leadership may interfere and influence priority setting and these setting and these other issues" (District level informant)*.

Contrary to formal decision-makers perspectives, community decision-makers and patients (53 of 65 informants and participants) perceived these differences in waiting time and services as discrimination.

*"Another issue is that they are so congregative [referring to discrimination practices]. They choose whom to attend to first. They always look, or judge at the outside appearance of some one, if you look dirty, the will not pay much attention to you"*. (participant: FGD with male adolescent)

### Access to medicines

Fairness relating to supply of medicines was reported by most formal decision-makers. In the public health system in Zambia, the Medical Stores is the only regulatory body mandated to procure drugs, and it supplies them at no cost to all public health facilities in the country. Thus cost and availability were not reported to be problematic from the decision-makers' point of view. It was common for procurement officers and those working in facilities to report adequacy of medicines. One informant said:

*"With my experience, like when it comes to drug allocations (including malaria drugs), you find it has always been more than 100%. Each year they will add more" *(District level decision-maker, male informant)

In cases that stock-out of medicines occurred, the procurement officers explained that they were able to buy medicines by borrowing money from local business people:

*"I know there are many challenges going back to the challenge of finances. There are times when there is inadequate finance because maybe we haven't received for two months or three months and we are not a profit - making organization. So we have created a rapporteur with businessmen who give us on credit through their credit facilities. So that's what we have been doing when we have that crisis of no money. In the district, we go to our partners, with whom we have developed that relationship with and they understand us" (*FGD with district level participant, male).

*"Ok, the rapporteur is not just with the local people here but also with the other providers of the drugs and other supplies. So mostly you find that the procurement officer will be requested to draw up the purchasing order and then make an order through those people (business stakeholders), so that the payment is done when funding comes" (*FGD district level participant, female).

In opposition to this, 49 of 52 patients complained about perpetual dispensary of malaria medicines by prescription instead of receiving the medicines free of charge. When asked, on average out of five visits made to the hospital, how often they would expect to be given prescriptions to go and buy anti-malarial drugs and panadols, the average response was three. There were strong suspicions that the health workers sold free drugs provided by Medical Stores to private chemists in order to personally gain money. One participant reported:

*"Like we walk about 2 hours from home and when you reach the clinic, you find that there are no medicines [referring to malaria medicines]. When the medicines come within 2 days, 3 days they are finished. In addition, those who give malaria medicines, many times, we are told to go and buy, they say they don't have drugs, go and buy panadols"*. (participant3, G9 schooling, 10 years length of residence in district: FGD with male outpatients 35 years and above)

### ITN distribution

The awareness and acceptance of ITNs as important in the prevention of malaria infection seemed to be high among the patients. When asked what strategy participants thought were most effective in preventing malaria, most (39 out of 52) of them mentioned ITNs. The main problem related to ITN use seemed to be access to subsidized ITNs according to formal decision-makers (10 of 25 informants), community decision-makers and patients (38 of 65 informants and participants). The DHMT was not supplied with ITNs or resources to buy these and could thus not distribute ITNs themselves, but had to rely on other stakeholders such as Society for Family Health, Care International and the District Director of Planning through District Water and Sanitation Health (D-WAHSHA) programme to supply and distribute ITNs. Thus the DHMT had little opportunity to ensure that the distribution was done according to the policy guidelines and the district activity plan. Although the DHMT indicated that the ITNs should primarily go to prioritized high risk groups and disadvantaged geographic areas, such as Lukanga swamps (fishing area in rural Kapiri-Mposhi), donors and international NGOs seemed to be content with increasing the overall coverage of ITNs and tended to distribute them in easily accessible areas in the centre of the district, and they did not control that vulnerable groups were prioritized during delivery. There were no mechanisms in place that ensured that malaria agents, neighbourhood health committees and community health workers did not sell ITNs outside their boundaries to people who could afford to pay. The District Director of Planning received ITNs from a number of different donors and tended to distribute these in areas where they were engaged in water and sanitation improvements, although these areas may not have been prioritized in the district health plans.

"We are not doing much concerning our primary prevention strategies, the use of ITN, sourcing of funds to purchase those ITNs, you know the spraying (In-door Residual Spraying in the district) and ensuring that we cut the grass around."

**"***Because there is funding for it *[referring to stakeholders' ability to supply and distribute ITNs] *and who ever is giving that money is saying that you work the way we want*. (District level decision-maker, male informant)

*"What can I say? I can say all is not well where the distribution of ITNs is concerned. You find that we are receiving just few nets and we are receiving them from the donors. I think it was going to be better if the government will come in and help on that because we need more mosquito nets and we need more retreatment kits for these nets. So you find maybe you receive just maybe 150 nets. After sometime, people (referring to antenatal mothers) will still be coming in. If you tell them to buy from the shops, then most of the time you find they will say, "we don't have money". At least the ones which we receive from the NGOs, they are buying at K3000" (District level decision-maker, female informant)*.

Patients indicated that access to subsidized ITNs in their opinion was inequitable as people holding administrative positions in the district seemed to have easier access. Rural areas were perceived to be neglected as the time needed to reach current distribution points for subsidized ITNs was regarded as unacceptable. In addition, health providers' were suspected of diverting subsidized ITNs to places where they could sell them at a higher price to personally make a bit of money (mentioned by 43 of 52 patients). They asked for mechanisms that ensured that suspected deviation of drugs and ITNs was investigated:

*"The problem here is that the ones who receive the mosquito nets *[referring to subsidized ITNs], *those in offices, you find they get many and they go to sell somewhere else"*. (participant4: FGD with male adolescents)

The problem of health workers selling ITNs was also known to the DHMT, and in order to minimize deviation of subsidized ITNs, the DHMT together with stakeholders supplying them, had put in place monitoring and verification mechanisms and processes. One informant responsible for distributing subsidized ITNs at facility level said:

*"The one who is in charge of the nets will go through what I have sold so that he should make sure that at least I have sold the things at the right price. I haven't removed anything from that money or I haven't done anything, all the nets, it's true I have sold to the mothers"*. (District level decision-maker, female informant).

Despite children under the age of five years on paper being prioritized for provision of malaria services and ITNs, patients perceived that they were not prioritized during delivery. There were also complaints that prioritized groups were not given appropriate care and treatment at health facilities (mentioned by all 28 female participants and 6 of 13 community decision-makers). One female participant narrated thus:

*"I brought my child who was very sick. So, I was told to go to room 12 after collecting the book. So, I went in there and as I was explaining to him that "the child is very sick", he even chased me out and allowed the people he knew well to go in first before me. But I just went into another room and also in there, he just gave me a prescription and said to go and buy. So like that, a child who is very sick can even die" *(participant3: G10 schooling, 6 months residence in district: FGD with female adolescents).

### Feedback

There was disagreement among informants and participants holding formal decision-making positions regarding the content of feedback relating to services being delivered. Whereas most decision-makers in this study (six out of nine participants in the DHMT FGD) perceived the community to be happy with the services offered based on the number of people accessing services, information obtained from letters to suggestion boxes placed at the facilities and the good comments the facility management and health staff received.

*"The community we are serving, many of them are appreciating the service. People have written letters appreciating what we have done, they are passing very good comments. For us we are saying, definitely we are doing fine with the communities that are working with us, yes" *(FGD with district level decision-makers, male participant)

However, there were a few (three out of nine participants) who admitted that patients expressed dissatisfaction. One participant reported how complaints received through suggestion boxes from the community should be handled according to national guidelines:

*"A leader should also be interested in getting feed-back from the community he or she is serving. Yes, we are saying whatever decision you make and take, you should check back and see is it having effect? Or is it being put into operation? Not where you are bulldozing, you don't know whether things are running or not (haa...laughs)*. (FGD with decision-makers at district level, female participant 2: 34 years old, 9 years of service).

*"And ordinary people are involved; there are suggestion boxes, and even interviews, which are done. So maybe in the suggestion box when they write something and then the hospital will open up the suggestion box, they will read, they wont feel offended when they have been insulted or whatever. And basing on what that particular person has written, it may cause management to make a decision. To say, "This is what the public is saying?" That we are starting our services late, can we improve? Nurses, now you will be reporting at such and such a time, you make a decision basing on what the public has said*" (FGD with district level decision-makers, male participant)

All the 52 participants who took part in the eight outpatient FGDs persistently called for improvements of appeal (feedback) mechanisms for complaints. One participant argued thus:

*"They should even tell people who to report to once you are shouted at. We always write in the suggestion box but there is no improvement"*. (participant 6, G8 schooling, 5 years residence in district: FGD with female adolescents).

### Legitimate stakeholders to involve during delivery

For decision-makers, legitimate stakeholders to involve in deployment and retention of health-providers included the District Medical Officer (DMO), health-providers, financial officers, human resource officers and public regulatory bodies *(civil service*). For supply of ITNs, donors and NGOs were the important stakeholders, whereas Medical stores of Zambia (*national parastatal institution*) and procurement officers were seen to be the legitimate stakeholders for supply of medicines, including malaria medicines. The social welfare office was reported as the stakeholder for support of stranded users (e.g elderly patients who travelled long distances to get to the hospital and, due to long waiting time to access services, were unable to return home and thus needed money for food and lodging).

*"Ok stakeholders apply through the Ministry of health. Then they will come down to the province and then we will be told (referring to the district level). If there are any changes, they will need to re-set the MOU (memorandum of understanding). For example, through the permanent secretary (PS) and the District Commission (DC) here (referring to political authorities) they agreed with the chief (referring to the local leadership) that these services could actually be taken to Chief YYY area (referring to geographic boundaries under the leadership of the local authority) The DC had to write and once that was done we were also informed. That was after the local leadership like the chief, and the DC at the district level and the PS had already agreed" *(District level decision-maker, male informant).

Patients added themselves, the indigenous healing system (healers), indigenous leadership (chiefs) and political position holders as stakeholders in all these processes, but did not agree that donors and NGOs were stakeholders at all. They thought that chiefs should have a role in monitoring the availability of drugs, subsidized ITNs and health personnel, and politicians were seen to be important as they were elected to represent the population's interests.

*"In this community, we have different leaderships. We have politicians who promised that they were going to bring good hospitals. Through complaining, because when things are not in position, I xxx (referring to patient himself/herself) sometimes face them. These people (referring to patients) most of the times are the ones taking their children to the hospital, who see whatever is going on. There are some people who don't even go to the hospital, they go to traditional healers. I am told there are some who are now full time with herbs *(Community level decision-maker, female informant).

The formal decision-makers did not recognize the community as legitimate stakeholders during health care delivery due to lack of medical or technical training, and they did not think that politicians or chiefs should be involved in the deployment and retention of health-providers for the same reasons. However, they reported that politicians did play an important role in practice in rural areas.

*"One staff *[referring to health-provider] *at one of the rural health centres, the chief took him to the provincial Permanent Secretary (PS). He didn't want him. A decision related to health, they are supposed to follow the channel, by seeing the District Director of Health (DDH), then let the civil service handle the issue, than the politicians or the chief" *(FGD with decision-makers district level, male participant).

During DHMT planning activities, rural community arms such as Neighbourhood Health Committees, malaria agents and Community Health Workers were included as legitimate stakeholders. However, in the actual distribution of ITNs by NGOs, the only community-based arm to be involved was the malaria agents. Patients complained about the lack of information on ITN distribution.

*"We have already said, the service for us in rural area, the information is not reaching the people. Like we hear that there are mosquito nets, sometimes we hear that in such an area they were selling at 10,000 or K3, 000, now this is another way to prevent for us who are out there and the hospital is far." *(participant 6, G1 schooling: FGD with male outpatients 15-34 years)

Table 3 shows identified aspects of unfairness and illegitimate decisions at many levels during delivery processes as perceived by patients and other community informants. The complaints have been systematized in accordance with the AFR conditions.

**Table 3:** Status of AFR conditions during delivery of malaria services and ITNs: Period 2006 to 2008

AFR condition	Explanation
**Relevance**	Reasons for deviating ITNs elsewhere were not given. Reasons for difficulties accessing available qualified health-providers and whether or not they were adequate were not given, neither were reasons for not prioritizing children <5 years old when delivering malaria services.

**Publicity**	One-way ineffective communication mechanisms regarding ITNs seemed to be in place. Feedback channels to support exchange of any information regarding malaria services were not identified.

**Revisions/appeals**	Non-responsive appeal mechanisms (suggestion box) for malaria services were reported. No appeal mechanisms during delivery of ITNs were identified.

**Enforcement**	Leadership was a monopoly of health staff and managers, who were not regarded as fair by many of the patients due to poor respect of them and poor response to their needs and demands. Policy guidelines and district activity plans for equitable ITN distribution and other malaria services were also poorly managed. Although the application of AFR was being introduced through the district health leadership, there was not yet evidence from patients and informal decision-makers of explicit enforcement of AFR conditions.

## Discussion

This study found that there were disagreements between formal decision-makers, patients and informal decision-makers regarding fairness during delivery of malaria services and distribution of ITN. The study also identified disagreements relating to identification of legitimate stakeholders to involve during delivery of malaria services and distribution of ITNs. The study applied the AFR approach to categorize perceptions, attitudes and practices which were used to judge fairness and legitimacy. This categorization revealed that patients repeatedly called for improvements relating to the AFR conditions at different levels of the healthcare service. Results showed that calls for improvements were strongest in the leadership condition, which was followed by relevance, whereas publicity and appeals shared an equal density. However, all the AFR conditions were poorly applied (Table 3).

Several studies have evaluated priority setting processes relating to malaria control activities and have recommended improvements [18-20], but they have described gaps in provision of malaria control activities in general from the global, continental and country-specific strategic planning perspectives. This is one of very few papers to describe, evaluate and recommend improvements for *"real-life" *priority setting processes relating to healthcare services and interventions at district level, facility [21] and community levels perspectives [9,22,23], and the first to focus on malaria services and ITN interventions.

### Fairness and legitimacy of healthcare services

The community participants in this study gave experiences of ill-treatment or differential treatment from individual health providers as evidence for unfairness in service delivery. Their impression was that differential treatment was so widespread that it was part of the system itself, although giving some people priority due to their position is a violation of ethical guidelines of the healthcare services. It is likely that implementation of a priority setting processes in the district based on clear and reasonable principles may also affect the behaviours of individual health providers, particularly if the priority setting process focuses on publicity and appeal mechanisms. Implementation of the AFR approach to priority setting within the district health system may facilitate social learning and result in more transparency and accountability at all levels of the health service delivery [24]. Studies on priority setting in health institutions that have used the AFR framework have presented similar findings which show the primary goal for healthcare institutions being able to provide healthcare services, regardless of any barriers or challenges at different levels of healthcare service [21,23,25]. Issues of fairness or legitimacy are considered less important.

Perceptions and definitions of fairness and legitimate decisions during utilization processes of malaria services and distribution of ITNs by study participants were in line with other definitions used within the AFR framework where "legitimacy referred to the moral authority of the people or institutions who exercise priority setting and how that authority is derived" [26]. Fairness on the other hand is within this framework defined as "a processes of making prioritization based on a balance between relevant wider stakeholder values and what is locally acceptable at a specific time and within a given context" [26]. Although formal decision-makers perceived decisions relating to delivery of malaria services to be fair and legitimate, the patients (community) did not. Data obtained from this study could not establish whether or not malaria services and ITN distribution were really inadequate as perceived by the study participants. However, it is well known that costs and geographic distance is a barrier to equitable utilization of malaria services in poor countries such as Zambia [27-29]. This was clearly illustrated when the utilisation of healthcare services in public health institutions declined after the introduction of cost-sharing policies in Zambia in the 1990 s [30]. Although malaria medicines dispensed within public health facilities and consultancy were free for all in rural areas, the frequent experience of receiving a prescription instead of medicines probably contributed to the widespread self-medication practices reported by participants. Self-medication has been found to contribute to impromptu and ineffective treatment of malaria infections [31]. Studies from several African countries have also found inequitable ownership and utilization of ITNs as indicated by participants in this study [32-38]. Notably, decision-makers did not report cost and or supply of malaria medicines as a hindrance to fair decision-making practices during delivery, probably because the Medical Stores of Zambia - a government parastatal institution was responsible for procurement, supply and distribution. Although stock out of medicines was not reported, it was indirectly referred to. It is possible that procurement officers together with departmental managers at the hospital and health centers were unwilling to admit that stock-out was a problem as they might have been afraid of being criticized themselves for not having requested or procured enough drugs from Medical Stores. Instead they tried to ease stock-out situations by borrowing money from local business people to buy medicines on the private market.

Whereas formal decision-makers based their evaluation of malaria services on technical perspectives and narrower values, the community combined both technical and non-technical criteria. In addition to technical criteria used by formal decision-makers, non-technical influences such as attitudes, adequacy of providers, accessibility to providers, medicines and subsidized ITNs, were also used by community decision-makers and participants. Formal decision-makers seemed to deny that some health-providers had problematic attitudes and behaviours towards patients, probably because they had few possibilities of sanctioning the health-providers, e.g dismissing them, due to recruitment and retention problems.

This study found that because of discriminatory practices by health-providers during delivery, influential people such as politicians, friends and family members of health-providers, those in formal employment etc seemed to have better access to malaria services and ITNs than the prioritized higher risk groups who came from lower socioeconomic positions. This study argues that prioritizing politicians during delivery could be due to conflicting characteristics used to judge a fair leader on one hand, and a fear of politicians instituting forced transfers to other rural areas and dismissals from jobs on the other. For the latter reason, health providers may have wished to make the time they spent at the facility as short as possible, so that their lack of knowledge and understanding did not interfere with progress relating to delivery of prioritized healthcare services.

Most of the patients in the FGDs reported experiences of bad treatment by health providers. This is likely to be related to the difficult conditions health-providers are working under. Understaffing, work overload, low salaries and long working hours may affect health workers' motivation for their job. In many settings in Zambia, health workers rely on receiving some kind of appreciations (e.g gifts or money) from supposedly satisfied patients. The health workers know that poor people have little opportunity to offer such material appreciation, and this may influence their attitude and behaviour towards these patients.

Although NGOs involved in ITN distribution agreed on prioritizing young children and pregnant women and certain areas at the planning stage, participants reported that the NGOs seemed to base their actual distribution on the principle that increasing coverage of ITNs in the general population was more important than focusing on certain vulnerable groups. In order to solve the problem of low access to subsidized ITNs for priority groups and areas, it thus seems that the DHMT needs to be given resources to distribute ITNs too or to establish control mechanism that can ensure that the nets distributed by other organizations/agencies, actually reach those that are prioritized in the guidelines and health plans. However, the distribution practice of the NGOs may be claimed to be justifiable too, and in line with the Ministry of Health policy statement *"to provide equity of access to cost effective, quality health care as close to the family as possible for all Zambian"*. Modelling indicates that the most equitable protection of vulnerable groups against malaria may be achieved by increasing ITN coverage in the whole population even to a modest level as this will reduce overall human-to-mosquito transmission of malaria parasites in the area and thus the risk of infection will decrease for all. With a high ITN coverage in the general population, even children and pregnant women who do not sleep under an ITN would be protected [39].

### Disagreements relating to legitimate stakeholders to involve during delivery

Whereas formal decision-makers reported legitimate stakeholders as those with technical knowledge and expertise within the field of health and who were working in public institutions, community decision-makers included non-technical persons such as politicians, chiefs, indigenous healers, and patients as legitimate stakeholders. Interestingly, rural community arms such as Community Health Workers, Neighbourhood Health Committees and malaria agents were considered as legitimate stakeholders during planning by formal decision-makers, but not by the community. This could be linked to the recruitment processes followed, where health-providers instead of communities themselves selected who should be involved in health activities.

Patients perceived themselves as legitimate stakeholders during delivery of healthcare services, and were appealing for recognition of this status. One way of involving the community in decision-making could be to call for meetings with the community when complaints in the suggestion boxes indicate serious problems with the health care services (as indicated in national guidelines). Anecdotal evidence from other parts of Zambia indicates that local communities may actually be able to offer solutions or provide assistance when problems arise in the health care system due to lack of resources and personnel. For example, volunteers may assist with caring for patients to allow health providers to focus on the more technically demanding tasks. Informing the community about the decreasing health budgets and reductions in clinic staff, may also increase patients' tolerance for the undesirable behaviour of exhausted staff members.

In order to reconcile the differences in perceptions of fairness of malaria services and ITN distribution, as well as to agree on which legitimate stakeholders to involve during delivery, an ethical framework such as AFR, may be of great help. This explicit framework promotes bringing together diverse stakeholders with varying values and principles to agree and, hence, take a decision perceived as fair and legitimate. Distribution of resources among competing health needs occurs at all levels (national, institutional, individual) and is a challenge all over the world. It is even more challenging in low income countries such as Zambia where the competition between and within health programmes and patients, could at times be extended to health-providers whose roles and concerns would differ. Decision-makers involved in priority setting processes should therefore aim for fair and legitimate decision-making processes in order to improve acceptability of decisions made. It has been argued that moral legitimacy of priorities does not just involve who has moral authority to set them, but how these priorities are set. Key elements of fair processes include transparency about the grounds for decisions; appeals to reasons that majority stakeholders can accept are relevant to meeting health needs fairly; and procedures for revising decisions in light of challenges to them. When participants in this study were asked about the presence or lack of equity, quality and trust related to service delivery and ITN distribution, they mentioned issues associated with relevance, publicity, appeals and leadership. Although these four terms were not necessarily employed, it was clear that the concepts were well known in the community and that the participants thought they were necessary to fulfill in order to achieve fairness and legitimacy. This is in line with the AFR framework which argues that decision-making processes can be improved in order to achieve legitimacy and fairness, since fair procedures involve empirically feasible practices that can be sustained and connect well with the goals of various stakeholders.

Although these results are based on data collected from a short time period (2006-2008) in one rural district of Zambia, the scarcity of resources in the health system, staffing problems, delayed allocations and limited sources for resources required to support effective and efficient provision of healthcare services, are common challenges, which districts all over the country share. This may make it difficult for districts to adhere to guideline recommendations relating to malaria services and distribution of subsidized ITNs in other districts in the country too. The shift in the priority setting related to malaria services and ITNs from top-down approach to a bottom-up approach at the district level is also very challenging. The members of the DHMT have medical and technical training, and it seems to be difficult for them to meaningfully involve the community during delivery without any skills in engaging the community. Thus the findings of this study are likely to be applicable in many other settings within Zambia.

## Conclusion

Poor status of the AFR conditions of relevance, publicity, appeals and leadership corresponds well to the differing perceptions of fairness and unfairness among outpatient attendees and decision-makers. This may have been re-enforced by existing disagreements between the two groups regarding who the legitimate stakeholders to involve during service delivery were. Conflicts identified in this study could be resolved by promoting application of approaches such as AFR during priority setting in the district.

## List of abbreviations

AFR: Accountability For Reasonableness; CHWS: Community Health Workers; DDP: District Director of Planning; DHMT: District Health Management Team; D-WASHA: District Water and Sanitation Health; GMCP: Global Malaria Control Programme; HCCS: Health Centre Committees; IRS: In-door Residual Spraying; ITNS: Insecticide-Treated Nets; MIM: Multi-lateral Initiative in Malaria; MSF: Medicine San Frontiers'; NHCS: Neighbourhood Health Committees; RBM: Roll Back Malaria; REACT: REsponse to ACcountable priority setting and Trust in health systems; SFH: Society for Family Health; WHO: World Health Organization.

## Competing interests

The authors declare that they have no competing interests.

## Authors' contributions

MT: Collected most of the data, conceived the idea of this paper, drafted the manuscript, analyzed data, interpreted, sole responsible for incorporating revisions, IFS: participated in interpretation and revisions, PB: Assisted in design and coordination of the study, participated in interpreting and revisions, JB: Conceived of the REACT study, overall coordination of this study, its design, data collection, participated in interpreting and revising this manuscript. All authors read and approved the final manuscript.

## References

[B1] Heggenhougen HK Hackethal V Vivek P (Eds.) The behavioural and social aspects of malaria and its control Geneva: UNDP/World Bank/WHO: Special Programme for Research & Training in Tropical Diseases (TDR) 2003 i x 1-215

[B2] Gamble C Ekwaru JP ter Kuile FO Insecticide-treated nets for preventing malaria in pregnancy (Review) The Conchrane Collaboration 2006 2 10.1002/14651858.CD003755.pub2 PMC6532581 16625591

[B3] World Health Organization Africa malaria report 2006 2006 Geneva. World Health Organization, Regional Offices for Africa and Eastern Mediterranean

[B4] Breman JG Alilio MS Mills A Conquering the intolerable burden of malaria: what's new, what's needed: a summary Am J Trop Med Hyg 2004 71 Suppl 2 1 15 15331814

[B5] Ministry of Health National Malaria Control Action Plan - Actions for Scale-up for Impact on Malaria In Zambia. Lusaka 2008

[B6] World Health Organization Roll Back Malaria 2005 Department operations support and capacity development. Geneva

[B7] Ministry of Health Sector Advisory Group Meeting Based on assessment of 3rd and 4th Quarter 2007 reports. Lusaka 2008

[B8] Tuba M Ngulube TJ Harmonization & Aid Effectiveness in Health: Between Health Workers & Communities: The Equity Gauge Zambia Experiences In 2nd Conference of the African Health Economics and Policy Associations: 26th-28th 2008 Livingstone, Zambia: Health Economics and Policy Network in Africa

[B9] Byskov J Bloch P Blystad A Hurtig A-K Fylkesnes K Kamuzora P Kombe Y Kvale G Marchal B Martin D Accountable priority setting for trust in health systems - the need for research into a new approach for strengthening sustainable health action in developing countries Health Res Policy Syst 2009 7 Suppl 1 23 10.1186/1478-4505-7-23 19852834 PMC2777144

[B10] Daniels N Sabin JE Accountability for reasonableness: an update BMJ 2008 337 a1850 10.1136/bmj.a1850 18845595

[B11] Daniels N Accountability for reasonableness BMJ 2000 321 1300 1301 10.1136/bmj.321.7272.1300 11090498 PMC1119050

[B12] Gruskin S Daniels N Process is the point: justice and human rights: priority setting and fair deliberative process Am J Public Health 2008 98 Suppl 9 1573 77 10.2105/AJPH.2007.123182 18633088 PMC2509612

[B13] Braveman P Gruskin S Theory and methods: Defining equity in health Journal of Epidemiology and Community Health 2003 57 254 8 10.1136/jech.57.4.254 12646539 PMC1732430

[B14] Central Statistical Office Summary report 2000 census. Lusaka 2003

[B15] Central Board of Health Health Institutions in Zambia: A Listing of Health Facilities According to Levels and Location for 2002. Lusaka 2002

[B16] Ministry of Health Selected HMIS Indicators for Districts: Second and Third level Hospitals: Quarter 4, 2006 to Quarter 4, 2007. Lusaka 2008

[B17] Moser CA Kalton G Social Methods in Social Investigations 1971 London: Heinemann Educational

[B18] Steketee RW Sipilanyambe N Chimumbwa J Banda JJ Mohamed A Miller J Basu S Miti SK Campbell CC National malaria control and scaling up for impact: The Zambia experience through 2006 Am J Trop Med Hyg 2008 79 45 52 18606763

[B19] Ngalame MP Williams AH Jones C Nyamongo I Diop S Gasper F Participation of African social scientists in malaria control: identifying enabling and constraining factors Malar J 2004 3 47 10.1186/1475-2875-3-47 15579214 PMC544396

[B20] Williams HA Jones C Alilio M Zimicki S Azevedo I Nyamongo I Sommerfeld J Meek S Diop S Bloland BP Greenwood B The contribution of social science research to malaria prevention and control Bull World Health Organ 2002 80 Suppl 3 251 2 11984613 PMC2567756

[B21] Reeleder D Martin DK Keresztes C Singer PA What do hospital decision-makers in Ontario, Canada, have to say about the fairness of priority setting in their institutions? BMC Health Serv Res 2005 5 8 10.1186/1472-6963-5-8 15663792 PMC548272

[B22] Kapiriri L Martin DK Priority setting in developing countries health care institutions: the case of a Ugandan hospital BMC Health Serv Res 2006 6 127 10.1186/1472-6963-6-127 17026761 PMC1609114

[B23] Kapiriri L Norheim OF Heggenhougen K Public participation in health planning and priority setting at the district level in Uganda Health Policy Plan 2003 18 Suppl 2 205 13 10.1093/heapol/czg025 12740325

[B24] Bandura A Social Learning Theory 1971 New York: General Learning Press

[B25] Bell JA Hyland S DePellegrin T Upshur RE Bernstein M Martin DK SARS and hospital priority setting: a qualitative case study and evaluation BMC Health Serv Res 2004 4 36 10.1186/1472-6963-4-36 15606924 PMC544195

[B26] Kapiriri L Martin KD A strategy to improve priority setting in developing countries Health Care Anal 2007 15 159 167 10.1007/s10728-006-0037-1 17922194

[B27] Mubyazi GM Bloch P Magnussen P Olsen OE Byskov J Hansen KS Bygbjerg IC Women's experiences and views about costs of seeking malaria chemoprevention and other antenatal services: a qualitative study from two districts in rural Tanzania Malar J 2010 9 54 10.1186/1475-2875-9-54 20163707 PMC2837674

[B28] Onwujekwe O Uzochukwu B Eze S Obikeze E Okoli C Ochonma O Improving equity in malaria treatment: relationship of socio-economic status with health seeking as well as with perceptions of ease of using the services of different providers for the treatment of malaria in Nigeria Malar J 2008 7 5 10.1186/1475-2875-7-5 18182095 PMC2249588

[B29] Uzochukwu BS Onwujekwe OE Socio-economic differences and health seeking behaviour for the diagnosis and treatment of malaria: a case study of four local government areas operating the Bamako initiative programme in south-east Nigeria Int J Equity Health 2004 3 Suppl 1 6 10.1186/1475-9276-3-6 15202941 PMC544024

[B30] Blas E Limbambala M User-payment, decentralization and health service utilization in Zambia Health Policy Plan 2001 16 19 28 11772987 10.1093/heapol/16.suppl_2.19

[B31] Kaona F Tuba M Improving ability to identify malaria and correctly use chloroquine in children at household level in Nakonde District, Northern Province of Zambia Malar J 2003 2 43 10.1186/1475-2875-2-43 14624700 PMC280690

[B32] Barat LM Palmer N Basu S Worrall E Hanson K Mills A Do malaria control interventions reach the poor? A view through the equity lens Am J Trop Med Hyg 2004 71 2 Suppl 174 178 15331835

[B33] Bernard J Mtove G Mandike R Mtei F Maxwell C Reyburn H Equity and coverage of insecticide-treated bed nets in an area of intense transmission of Plasmodium falciparum in Tanzania Malar J 2009 8 65 10.1186/1475-2875-8-65 19371415 PMC2674468

[B34] Matovu F Goodman C Wiseman V Mwengee W How equitable is bed net ownership and utilisation in Tanzania? A practical application of the principles of horizontal and vertical equity Malar J 2009 8 109 10.1186/1475-2875-8-109 19460153 PMC2695473

[B35] Noor AM Amin AA Akhwale WS Snow RW Increasing coverage and decreasing inequity in insecticide-treated bed net use among rural Kenyan children PLoS Med 2007 4 e255 10.1371/journal.pmed.0040255 17713981 PMC1949846

[B36] Onwujekwe O Hanson K Fox-Rushby J Inequalities in purchase of mosquito nets and willingness to pay for insecticide-treated nets in Nigeria: challenges for malaria control interventions Malar J 2004 3 6 10.1186/1475-2875-3-6 15023234 PMC395839

[B37] Onwujekwe O Malik el F Mustafa SH Mnzavaa A Do malaria preventive interventions reach the poor? Socioeconomic inequities in expenditure on and use of mosquito control tools in Sudan Health Policy Plan 2006 21 Suppl 1 10 16 16301307 10.1093/heapol/czj004

[B38] Webster J Lines J Bruce J Armstrong Schellenberg JR Hanson K Which delivery systems reach the poor? A review of equity of coverage of ever-treated nets, never-treated nets, and immunisation to reduce child mortality in Africa Lancet Infect Dis 2005 5 Suppl 11 709 717 10.1016/S1473-3099(05)70269-3 16253888

[B39] Killeen GF Smith TA Ferguson HM Mshinda H Abdulla S Lengeler C Kachur SP Preventing childhood malaria in Africa by protecting adults from mosquitoes with insecticide-treated nets PLoS Med 2007 4 Suppl 7 e229 10.1371/journal.pmed.0040229 17608562 PMC1904465

